# Recurrent neural network model of gait can predict Parkinson’s disease

**DOI:** 10.1093/bib/bbaf631.078

**Published:** 2025-12-12

**Authors:** Rishav Selvam

## Abstract

**Background:**

Parkinson’s disease (PD) is a progressive neurodegenerative disorder characterized by motor and non-motor symptoms, with subtle gait alterations often appearing in early stages. Early detection is critical for timely intervention, yet current clinical methods are limited. Wearable sensors offer the ability to capture high-resolution gait data, providing an opportunity to detect PD before pronounced clinical symptoms emerge.

**Methodology:**

In this study, we analyzed gait data from 166 participants, including 93 PD and 73 controls, collected using eight force sensors per foot at 100 Hz during a 120-second walking task. Force measurements were normalized using Min–Max scaling to reduce variability due to body weight and individual differences. Walking trials were segmented into 200-sample sliding windows, and a recurrent neural network (RNN) was trained to classify participants based on temporal patterns in gait dynamics.

**Results:**

The RNN accurately (96.4% accuracy) distinguished PD patients from healthy controls, capturing subtle differences in gait dynamics that are not apparent to clinicians.

**Conclusions:**

Overall, these findings demonstrate the potential of combining wearable sensors with machine learning for non-invasive, objective detection of Parkinson’s disease. Future work should validate the model on independent cohorts and explore its applicability in real-world settings to support earlier interventions and improve patient outcomes.

**References:**

1. “Statistics.” *Parkinson’s Foundation*.

2. Haaxma CA, Bloem BR, Borm GF, et al. “Gender differences in Parkinson’s disease.” *J Neurol Neurosurg Psychiatry* 2007; 78:819–824.

3. Wright Willis A, Evanoff BA, Lian M, et al. “Geographic and Ethnic Variation in Parkinson Disease: A Population-Based Study of US Medicare Beneficiaries.” *Neuroepidemiology* 2010; 34:143–151.

4. Dauer W, Przedborski S. “Parkinson’s disease: mechanisms and models.” *Neuron* 2003; 39:889–909.

5. Zhou ZD, Yi LX, Wang DQ, et al. “Role of dopamine in the pathophysiology of Parkinson’s disease.” *Transl Neurodegener* 2023; 12:44.

6. Gandhi KR, Saadabadi A. “Levodopa (L-Dopa).” *StatPearls* 2025.

7. Wang J, Hoekstra JG, Zuo C, et al. “Biomarkers of Parkinson’s disease: current status and future.” *Drug Discov Today* 2013; 18:155–162.

8. Lehéricy S, Sharman MA, Dos Santos CL, et al. “Magnetic resonance imaging of the substantia nigra in Parkinson’s disease.” *Mov Disord* 2012; 27:822–830.

9. Morris R, Hickey A, Del Din S, et al. “A model of free-living gait: A factor analysis in Parkinson’s disease.” *Gait Posture* 2017; 52:68–71.

10. Hausdorff JM. “Gait in Parkinson’s Disease.” *PhysioNet* 2008.

